# Cup versus bottle feeding for hospitalized late preterm infants in Egypt: A quasi-experimental study

**DOI:** 10.1186/1746-4358-3-27

**Published:** 2008-11-21

**Authors:** Amel M Abouelfettoh, Donna A Dowling, Soheir A Dabash, Shadia R Elguindy, Iman A Seoud

**Affiliations:** 1 Francis Payne Bolton School of Nursing, Case Western Reserve University, Cleveland, Ohio, USA; 2 Department of Pediatric Nursing, Faculty of Nursing, Cairo University, Cairo, Egypt; 3 Faculty of Medicine, Cairo University, Pediatric University Hospital, Cairo, Egypt

## Abstract

**Background:**

Although previous studies have demonstrated beneficial breastfeeding outcomes when cup feeding rather than bottle feeding was used for feeding preterm infants, cup feeding has not been implemented in Egypt. The aim of the current study was to examine the effect of using cup feeding as an exclusive method of feeding preterm infants during hospitalization on breastfeeding outcomes after discharge.

**Methods:**

A quasi-experimental design, with the control group studied first, was used to examine the effect of cup feeding for preterm infants on breastfeeding outcomes after discharge. Sixty preterm infants (mean gestational age was 35.13 weeks and mean birth weight was 2150 grams) were recruited during Neonatal Intensive Care Unit (NICU) stay. Control group infants (n = 30) received only bottle feedings during hospitalization and the experimental group (n = 30) received only cup feedings during hospitalization. Both groups were followed up after discharge for six weeks to evaluate infant's breastfeeding behavior and mother's breastfeeding practices. Data were analyzed using descriptive statistics and repeated measures ANOVA for testing the differences between the cup feeding and bottle feeding groups over six weeks after discharge.

**Results:**

Cup fed infants demonstrated significantly more mature breastfeeding behaviors when compared to bottle fed infants (p < 0.01) over six weeks, and had a significantly higher proportion of breast feedings one week after discharge (p = 0.03).

**Conclusion:**

Cup fed infants were more exclusively breast fed one week after discharge, supporting the Baby Friendly Hospital Initiative recommendations for using cup feeding and avoiding bottle feeding when providing supplementation for preterm infants. The current study provides initial evidence for the implementation of cup feeding as a method of supplementation for late preterm infants during hospitalization.

**Trial Registration:**

Clinical Trial NCT00756587.

## Background

The provision of breast milk is essential for preterm infants as it provides unique health benefits that are unmatched by other types of feeding [1-3]. However, breastfeeding presents unique challenges for preterm infants that include establishing and maintaining the mothers' milk supply and transitioning the infant from gavage feeding to breastfeeding [4]. One of the issues that presents during the transition to breastfeeding is that mothers of preterm infants are rarely available for all oral feedings during hospitalization; making it necessary for infants to receive oral feedings by other methods, usually bottle feeding.

However, exposure of newborn infants to artificial nipples has been strongly associated with breastfeeding problems [5-9]. Frequently these problems have been explained by a phenomenon called nipple confusion. Nipple confusion occurs when infants are exposed to two different feeding methods, bottle and breast, resulting in the infant refusing to breastfeed [10]. Consequently, it has been recommended that bottle feeding be avoided and that cup feeding be used for the supplementation of term [11,12] as well as preterm infants [13-15].

Cup feeding is known as an alternative method of feeding breast milk to an infant using a small cup without a lip [16,17]. Cup feeding is also recommended by the Baby Friendly Hospital Initiative [18]. The use of the cup for feeding newborn infants was originally based on the goal of avoiding propping up of bottles and also to increase bodily contact with the mother during feeding [19]. Although cup feeding receives little mention in medical literature, and may seem to be a new technique for some, cup feeding has been used in several developing and developed countries [16]. Lang, who observed cup feeding in South Nepal, implemented cup feeding in England and the practice expanded to other developed countries. Consequently cup feeding was established as a method for feeding infants who could not be breastfed from birth [15].

The findings of studies concerning breastfeeding outcomes of cup fed infants have been inconsistent. A Cochrane review [20] concluded that cup feeding cannot be recommended over bottle feeding as a supplement to breastfeeding because cup feeding had no significant benefit in maintaining breastfeeding beyond hospital discharge. Also, the review suggested that cup feeding had the potential for the unacceptable consequence of a longer hospital stay. A randomized controlled trial [21] compared the impact of cup or bottle supplementation for preterm infants on subsequent breastfeeding at discharge from the hospital. No significant differences were found between the bottle and cup feeding infants in terms of whether they were breastfeeding or not at discharge from the hospital. However, the small sample size (n = 12) may have contributed to the lack of differences. In contrast, another report suggested that infants in special care units who are supplemented by cup are more likely to breastfeed longer than those supplemented by bottle [22].

Cup feeding has not been implemented in Egypt, making evaluation of its use essential. Additionally, given the absence of definitive evidence as to the most effective method of supplementation for preterm infants during hospitalization and the effects of cup feeding on breastfeeding patterns after hospital discharge, the purpose of the current study was to examine the effect of cup feeding on breastfeeding in late preterm infants after discharge. The following research questions were addressed: (1) Are premature infants supplemented by cup during hospitalization more likely to be fully breastfed six weeks after discharge when compared to premature infants supplemented by bottle during hospitalization?, and (2) Do preterm infants supplemented by cup during hospitalization demonstrate more mature breastfeeding behavior at 1, 2, 3, 4, 5, and 6 weeks after discharge when compared to preterm infants supplemented by bottle?

## Methods

### Design

A quasi-experimental cohort design was employed using two groups. The first group, the control (bottle) group, received all oral feedings by bottle during hospitalization as that was the standard practice in the Neonatal Intensive Care Units (NICUs) where the study was conducted. The control group was studied first to avoid the exposure of the control group to the intervention, cup feeding. The second group, the intervention (cup) group, received all oral feedings by cup during hospitalization. Infants in both groups were studied weekly for six weeks after discharge.

### Sample

**Figure 1:**
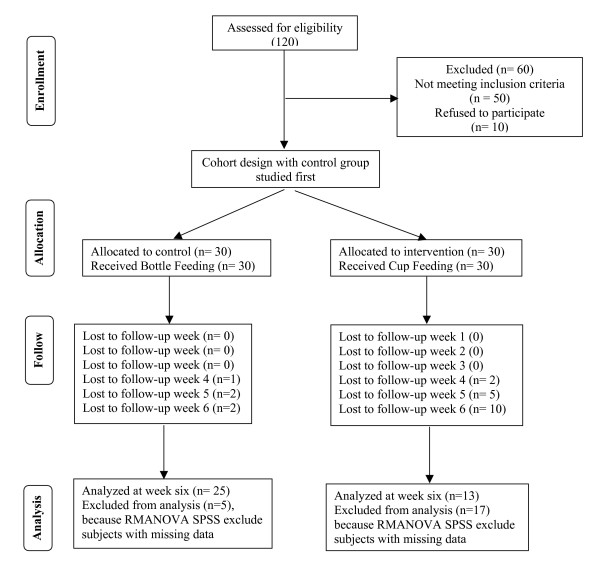
Sampling flow chart.

The convenience sample consisted of 60 late preterm infants admitted to the NICU. Thirty infants were assigned to the control group and the next 30 to the intervention group. To calculate the sample size, statistical power analysis was performed using a medium effect size and a power of 80% [23]. Breastfeeding prevalence at discharge from a previous study [14] was used to conduct the power calculation. Infants met the following inclusion criteria: (a) singleton birth, (b) 34 to 37 weeks of gestation at birth, (c) maternal intention to breastfeed, (d) no supplemental oxygen required, and (e) being fed only by intermittent gavage feeding at the time of recruitment. Infants could be in open cribs, radiant warmers, or incubators. Infants who had any condition interfering with oral feeding, including an oral congenital anomaly, intracranial hemorrhage, and/or craniofacial anomalies, were excluded. All potentially eligible infants and mothers were approached sequentially until the required sample was completed for each group, with the intervention group being recruited after completion of the control group. Total attrition for the study was 22 mothers, with one mother not returning for the fourth week visit, nine more mothers not returning at week five and an additional 12 mothers were lost at week six. At week six, 25 mothers in the control group and 13 mothers in the intervention group remained in the study (Figure 1).

### Setting

The study was conducted in two transitional nurseries in neonatal intensive care units at Pediatric University Hospital, Cairo, Egypt. Mothers in the current study setting were not instructed on how to express breast milk.

### Instruments

Two study instruments were used for data collection. The first was The Maternal Breastfeeding Practice Questionnaire (MBP), developed for use in this study to assess daily infant feeding practices during the past week. The MBP included demographic questions as well as questions that assessed breastfeeding frequency and the number of bottle feedings, and whether artificial formula or any other type of feedings had been given to the infant. The proportion of feedings that were breastfeeding (direct breastfeeding or any expressed breast milk) was classified according to the Labbok and Krasovec schema for the definition of breastfeeding [24]. The schema divides the act of breastfeeding into three major categories: full breastfeeding (exclusive and almost exclusive), partial breastfeeding, and token breastfeeding. Exclusive breastfeeding means that nothing other than breast milk enters the infant's mouth. Almost exclusive breastfeeding means that water, vitamins or ritualistic feedings like herbal drinks are given infrequently but not for nutritional purposes. Partial breastfeeding means supplementing the infant's feedings with other foods or liquids, and includes three levels: "High," "Medium," and "Low". Partial breastfeeding levels represent the proportion of breastfeedings per day, or the relative amount of breast milk consumed to any other feeds (> 80%, 20 – 80%, < 20%). Token breastfeeding reflects minimal and irregular breastfeeding that constitute less than 15% of the total daily feedings, and using the breast primarily for infant comfort and consoling, not for nutrition.

The Premature Infant Breastfeeding Behavior Scale (PIBBS) [25] was the second instrument. The PIBBS was used to measure the infant's breastfeeding behaviors at one to six weeks after discharge. The PIBBS consists of 11 items; six of these items measure the development of preterm infant's breastfeeding behavior, while the other five items measure factors related to the breastfeeding session, such as the infant's general behavior, presence of letdown reflex, how long the infant was held, presence of any breast problem, and influence of the environment. Consequently, only the six items used for the scoring of the infant's breastfeeding behavior were used in the current study. The items were rooting, areola grasp, longest duration of latching, amount of sucking, longest sucking burst, and swallowing [25]. Face validity of the PIBBS was determined by three experts working in a WHO project on breastfeeding and demonstrated good capacity to describe maturational steps in infant breastfeeding behavior, ranging from the most immature to full term mature behavior. Inter-rater reliability of the PIBBS showed acceptable and satisfactory agreement between two observers in terms of percent of agreement and Kappa values (0.88, 0.72) respectively [26]. For the current study, reliability of the PIBBS was measured and showed good reliability (Cronbach's alpha based on standardized items 0.88).

### Procedures

The study was approved by the research committee at the School of Nursing, and the Pediatric University Hospital, Cairo University. The principal investigator (PI) reviewed the eligibility of each infant admitted to the transitional nursery during December 2003 to August 2004. If the infant met the eligibility criteria, the study was described to the mother. Mothers' verbal consent was obtained before data collection which was the standard procedure for consent in that setting. For both groups, oral feedings were started when determined by the attending physician in the NICU. Infants were fed either by bottle (control group) or by cup (intervention group) from the time oral feeding was started until discharge. Bottle feedings were given either by the assigned nursing staff or by the PI. All cup feedings were given by the PI or by one of two research assistants who are staff nurses at the NICU and who had been trained in the cup feeding technique by the PI. Lang's cup feeding technique [17] was used.

After infants were discharged from the NICU, mothers were interviewed at the first outpatient visit (one week post discharge) in a private room adjacent to the NICU to recall their breastfeeding practices during the previous week. Additionally, mothers and infants were observed by the PI during one breastfeeding session weekly for six weeks for assessment of infants' breastfeeding behaviors. The observation unit was a breastfeeding session defined as beginning when the mother initiated skin-to-skin contact with her infant and ending when skin-to-skin contact was terminated. The PI sat near the mother in a position which provided the best possible visibility of the infant's face and chin, and the infant's behavior at the breast was recorded using the PIBBS. At the end of the first breastfeeding session the PI asked the mothers the questions included in the Maternal Breastfeeding Questionnaire. It included questions about the frequency of breastfeeding during the day and the night, if any bottle feedings were given to the infant since discharge from the hospital, what was given (i.e. infant formula) and the frequency.

### Statistical analysis

Data were analyzed using the Statistical Package for the Social Sciences (SPSS) version 14. Descriptive statistics (frequency, percentage, mean, standard deviation, and range) were used to describe demographic characteristics of infants and mothers. Differences between groups for demographic interval data were determined using chi-square and for continuous data using t-tests. Two-way repeated-measures ANOVA was used to examine the effect of feeding method (between-subjects effect) and time (within-subjects effect) and the interaction between feeding method and time on the preterm infant breastfeeding behaviors after discharge. A Type I error of 0.05 was used as the level of statistical significance for all tests.

## Results

Demographic characteristics for the cup and bottle feeding infants and their mothers are presented in Table 1. For the entire sample the mean duration of hospitalization was 17.5 days (Standard Deviation (SD) = 9.1 days) with no significant difference between cup and bottle groups. Infants had few direct breastfeeding experiences during hospitalization that ranged from one to ten times (mean = 2.8, SD = 3.1) and a mean duration of cup or bottle feeding of 9.1 (SD = 5.6 days) and 12.5 days (SD = 8.2 days) respectively with no statistically significant differences between the two groups for their breastfeeding experience or days on cup or bottle feedings during hospitalization.

**Table 1:** Comparison of demographic characteristics of infants and mothers

**Characteristic**	**Bottle group** **N = 30** **mean (SD)**	**Cup group** **N = 30** **mean (SD)**	**t**	**p**
Gestational age at birth (wks)	35.3 (1.1)	34.9 (0.9)	1.27	0.23
Gestational age at discharge (wks)	38.1 (1.2)	37.2 (0.9)	3.16	< 0.01
Birth weight (grams)	2033 (329)	2267 (319)	2.78	< 0.01
Days in hospital	19.4 (9.8)	15.5 (8.1)	1.69	0.09
Days using cup or bottle	12.5 (8.2)	9.1 (5.6)	1.85	0.06
Mothers' age	26.5 (5.2)	27.3 (6.1)	0.55	0.58
Numbers of breastfeeds in hospital	2.4 (2.9)	3.2 (3.3)	0.99	0.33

	**Bottle group** **N = 30** **n (%)**	**Cup group** **N = 30** **n (%)**	***x***^2^	**p**

**Mothers' education**			0.38	0.83
No education	8 (27)	8 (27)		
Some education	8 (27)	10(33)		
Educated	14 (46)	12 (40)		
**Mothers' occupation**			0.07	0.57
Not working	20 (67)	23 (77)		
Working	10 (33)	7 (23)		
**Delivery**			0.07	0.39
Vaginal	13 (43)	12 (40)		
Caesarian section	17 (57)	18 (60)		
Previous breastfeeding	16 (53)	15 (50)	0.07	0.79
Previous bottle feeding	11 (37)	12 (40)	0.07	0.79

### Research question 1

Are premature infants supplemented by cup during hospitalization more likely to be fully breastfed (directly at breast and/or given expressed breast milk) six weeks after discharge when compared to premature infants supplemented by bottle during hospitalization?

Determination of breastfeeding practices would have continued for six weeks after discharge according to the design but, long term documentation was not feasible because 56% of the mothers had either some or no education. Low education also created concerns regarding accuracy of maternal verbal recall. Consequently, breastfeeding practices only for the first week after discharge are reported. The overall mean proportion of feedings that were breastfeeding (direct breastfeeding or provision of expressed breast milk) one week after discharge was 72%, with significantly higher proportion occurring in the cup feeding group (mean = 80, SD = 25.69) than in the bottle feeding group (mean = 64.4, SD = 29.50) (t = 2.22, p = 0.03) (Table 2).

**Table 2:** Feeding practices one week post discharge

**Feeding practices**	**Bottle group** **N = 30** **n (%)**	**Cup group** **N = 30** **n (%)**	**t**	**p**
Proportion of breastfeeding	64.4 (29.5)	80.2 (25.7)	2.22	0.03
No. of breastfeeds/day	6.8 (3.4)	8.5 (3.1)	2.07	0.04
No. of bottle feeds/day	3.6 (3.0)	1.8 (1.9)	2.79	< 0.01

**Bottle feeding**	20 (56)	16 (44)	1.11	0.22
Formula	15 (75)	7 (44)		
Expressed breast milk	1 (5)	0 (0)		
Ritualistic feeds	3 (15)	9 (56)		

**Figure 2:**
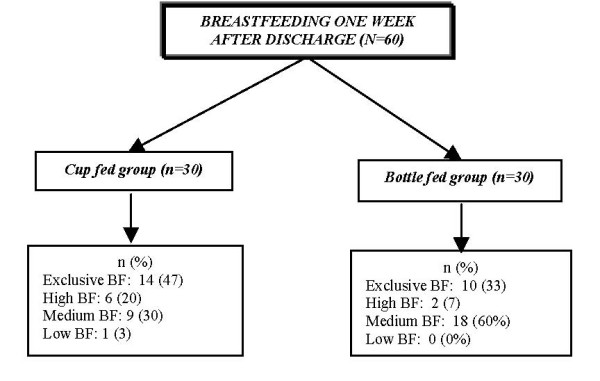
Daily feeding type one week post-discharge.

Figure 2 depicts the exclusivity of breastfeeding (direct breastfeeding and expressed breast milk) for infants in both groups one week after discharge. More infants in the cup feeding group were almost exclusively breastfed at one week after discharge when compared to infants in the bottle fed group (47% & 33% respectively). However, no statistically significant differences between the two groups in relation to the type of breastfeeding (either exclusive breastfeeding or partial breastfeeding were found (χ^2 ^= 1.1, p = 0.29).

### Research question 2

Do premature infants supplemented by cup during hospitalization demonstrate more mature breastfeeding behavior at 1, 2, 3, 4, 5, and 6 weeks after discharge when compared to premature infants supplemented by bottle?

**Figure 3:**
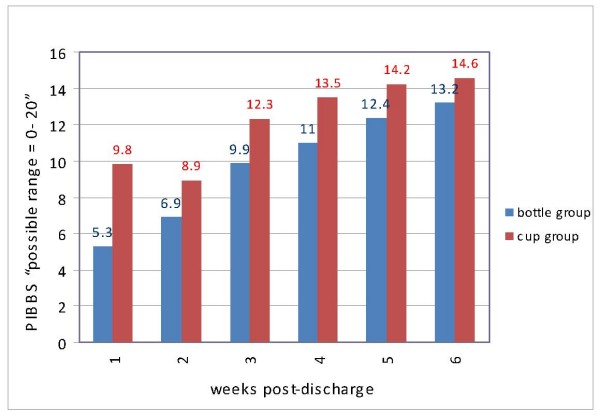
PIBBS mean scores over six weeks post-discharge.

**Figure 4:**
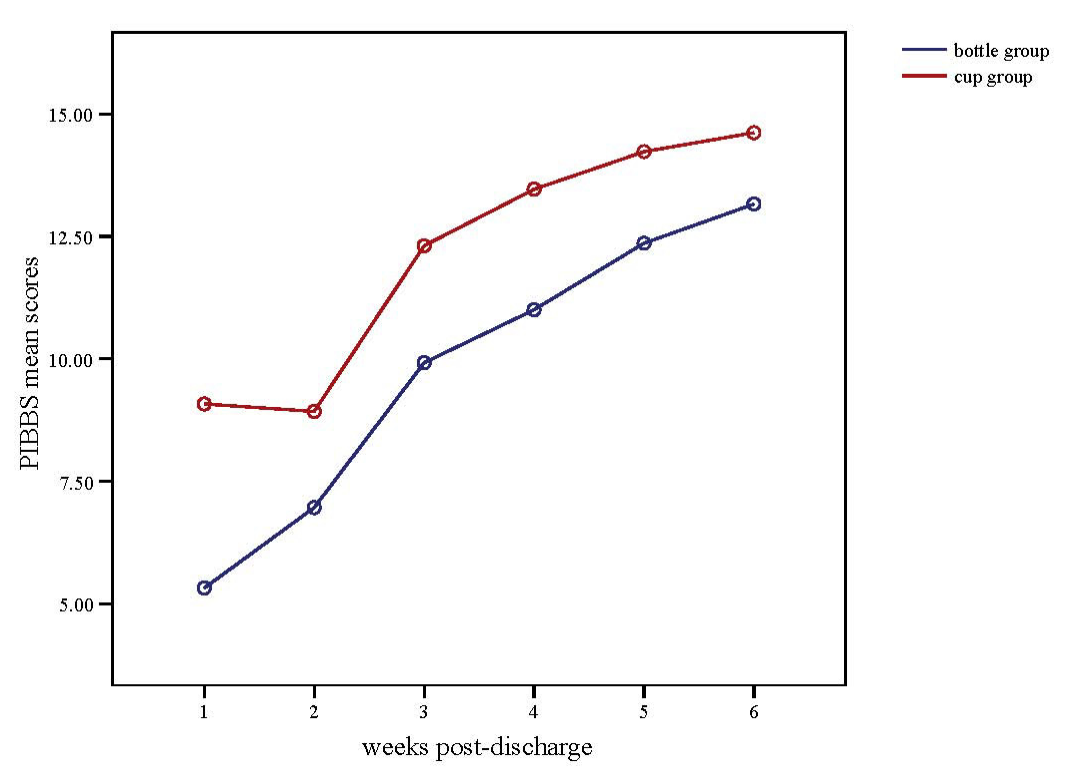
Group and time interaction of PIBBS mean scores over six weeks post-discharge.

Maturation of breastfeeding behavior was measured using the PIBBS. Figure 3 demonstrates the mean PIBBS scores for the both groups from week one through week six. Statistically significant differences in infant's age at discharge existed between infants in the cup feeding and bottle feeding groups, reflecting the younger age of infants in the intervention (cup feeding) group (t = 3.16, p < 0.01) (Table 1). Infants in the cup feeding group had a higher PIBBS score than infants in the bottle feeding group from the first week after discharge through the sixth week after discharge. Repeated-measures analysis of variance, using the general linear model (GLM REPEATED), was used to examine the differences in the infants' breastfeeding behavior over time for the two groups. Because the assumption of compound symmetry was not met, the multivariate results (Wilks' Lambda) are reported [27] (Table 3). An overall statistically significant difference in PIBBS Score existed between the bottle and the cup feeding groups, with the higher PIBBS scores occurring in cup feeding group (p = < 0.01). Also, there was a statistically significant difference for time effect (over six weeks after discharge) (p < 0.01), reflecting increasing scores for both groups across the six time points. The interaction between group and time was also statistically significant (p = 0.04). This interaction is presented in Table 3 and illustrated Figure 4.

**Table 3:** Repeated measures analysis for the PIBBS scores over six weeks post-discharge

**Variable**	**Factor**	**F**	**df**	**p**
PIBBS scores	Group	11.86	1.00	< 0.01
	Time	157.90	2.47	< 0.01
	Group*time	3.21	2.47	0.04

## Discussion

Findings of the current study should be interpreted in the light of the following limitations. First, a randomized experimental design could not be used because the introduction of cup feeding would have been a threat to internal validity through the possible diffusion of cup feeding to the control group. Consequently, randomization was not possible. Second, the principal investigator collected all of the data, including the assessments of breastfeeding behavior after discharge. Thus, the PI was not blinded to group assignment or to the purpose of the study. Further study using independent data collectors who are blind to group assignment and purpose is required to overcome these limitations. Third, it was intended that determination of breastfeeding practices would have continued longer after discharge. However, 56% of the mothers had either only some or no education so that long term written documentation was not feasible. Additionally, concerns regarding accuracy of maternal verbal recall were present. These concerns were minimized by recording breastfeeding practices for the first week after discharge only. Use of a simple feeding diary is recommended for future studies. Finally because of the loss to follow up, the sample was too small to adequately answer the primary research question. Future research needs to be planned using a larger sample size to account for attrition. Despite the limitations, the current study was the first to implement cup feeding for preterm infants in Egypt and was one of the few studies to use cup feeding as the only oral feeding method for preterm infants during hospitalization [5,13,14].

All 60 mothers intended to breastfeed after discharge. Even though, only 17 infants in the bottle feeding group and 20 infants in the cup feeding group had breastfeeding experiences during hospitalization. The low incidence of breastfeeding during hospitalization is most likely a result of many factors, including infrequent visiting and lack of encouragement of the mothers to be actively involved in their infants' care during visitation. Consequently, 38% of the infants were discharged with no breastfeeding experiences during hospitalization. Despite this, the overall mean proportion of feedings that were breast feeding for the entire group one week after discharge was 72%, with a significantly higher proportion in the cup feeding group when compared to the bottle feeding group. Infants in the cup feeding group had significantly more breastfeedings per day one week after discharge from the hospital than infants in the bottle feeding group, suggesting that the transition to breastfeeding progressed more quickly for cup feeding infants than for bottle feeding infants. The lack of exposure of cup fed infants to oral mechanisms used during bottle feeding, which are different than the oral mechanisms used during breastfeeding, [17] might facilitate adaptation to breastfeeding. However, the explanation for this finding is unclear [28].

Sipping and lapping used during cup feeding has been theorized to enhance development of tongue movements needed for breastfeeding [17]. However, the mechanisms of sipping and lapping differ from those required during breastfeeding [7,28]. Sipping and lapping require the lips to be closed, rather than open, as required during breastfeeding and to a lesser extent during bottle feeding. Differences in mouth contour, activity of the masseter, temporalis and buccinators muscles, and the position of the lips between cup, bottle and breastfeeding may contribute to subsequent breastfeeding difficulties [29]. A recent electromyographic study carried out during cup feeding, bottle feeding and breastfeeding found that the range of contraction and mean contraction of the masseter muscle were greater during cup feeding than during bottle feeding [10]. This finding supported the recommendation that if breastfeeding is not possible at certain times, cup feeding may be indicated, as it allows the participation of the masseter and temporalis muscles in a way that is similar to the participation of these muscles during breastfeeding [10]. These differences in oral mechanisms underlie the differences found in breastfeeding patterns when alternative methods such as bottle feeding have been used for supplementation of the breastfeeding infant.

An increased prevalence of breastfeeding has been reported when bottle feeding was replaced by cup feeding for preterm infants [17,20] as well as full term infants [12]. The findings of the current study are consistent with a recent randomized controlled trial [14] that found that cup feeding significantly increased the odds of breastfeeding at discharge. Additionally, Collins et al. reported a significant increase in the prevalence of breastfeeding at three and six months after discharge for infants fed by cup during hospitalization when compared with bottle supplementation [13]. In contrast, another randomized controlled trial provided unclear evidence of the effect of using cup or bottle for feeding preterm infants on breastfeeding [14]. No differences in breastfeeding prevalence at the first return visit between infants fed by cup and infants fed by bottle during hospitalization were found. At the first visit, 56% of bottle fed infants and 57% of cup fed infants had already been weaned and both groups presented similar breastfeeding prevalence. However, the percentage of infants still breastfeeding was two times greater in the cup fed group.

Although there were no statistically significant differences in the current study between cup feeding and bottle feeding groups regarding their breastfeeding type (full or partial) one week after discharge, more infants were exclusively breastfed in cup feeding group than in the bottle feeding group (Figure 2). These results are consistent with previous cup feeding studies [13,19] that reported that cup feeding significantly increased the likelihood that the preterm infants would be fully breast fed at hospital discharge. Additionally, it has been demonstrated that exclusivity of breastfeeding at one month after birth predicted the likelihood of continuing breastfeeding at six months [30]. In the current study it is possible that the shorter duration of cup feeding (9.1 days ± 5.61) than bottle feeding (12.5 days ± 8.20) resulted in these more optimal findings, as infants in the cup feeding group had less exposure to a feeding method other than breastfeeding. However, these findings were not significantly different, suggesting that it is the process of cup feeding rather than the duration of the feeding method that contributed to the better breastfeeding for the cup feeding group. In contrast to the suggestion of a recent Cochrane review [20], the length of hospital stay was shorter for the cup fed infants.

The second research question examined the maturation of breastfeeding behaviors from one through six weeks after discharge for both cup and bottle groups. The study findings demonstrated statistically significant differences between the cup and bottle feeding groups in their total breastfeeding behavior scores from the first-to-the sixth week after hospital discharge, reflecting higher mean PIBBS scores at each time point for the cup fed infants than the bottle fed infants. Additionally, infants in both groups showed an increase in their PIBBS scores over the six weeks, indicating a maturation of breastfeeding behavior over time. These findings are consistent with those of Nyqvist [31].

There was a significant interaction effect between group and time, demonstrating that, although both groups demonstrated expected maturation of breastfeeding behaviors, cup fed infants were significantly more mature in their breastfeeding behaviors at all time points than bottle fed infants, despite the cup feeding infants having statistically significant younger ages at discharge. The finding of improved breastfeeding behavior maturation among cup fed infants may be related to the higher breastfeeding proportion for this group, in that more breastfeeding experience may promote the maturation of breastfeeding behaviors. Conversely, more mature breastfeeding behavior may promote the frequency of breastfeeding.

Most previous studies have been concerned only with descriptions of infants' sucking and swallowing behavior [30], physiologic responses [32,33], and milk transfer [34]. Nyqvist, (1996) developed the PIBBS and used the instrument to describe the behaviors of breastfeeding preterm infants. However, the current study is the first to use the PIBBS to compare groups of breastfeeding preterm infants in relation to the method of supplementation [25]. A recent report used the PIBBS to evaluate breastfeeding behaviors for two groups of term infants to determine if epidural anesthesia had an effect on breastfeeding behaviors. There were no statistically significant differences found between the groups [35]. However, the PIBBS was not an appropriate instrument for use in that study as it had been developed for use with preterm infants. Future research should focus on the use of the PIBBS to compare preterm infants in relation to a variety of different experiences this population may have during the transition to full oral feeding.

## Conclusion

Cup fed infants were more exclusively breast-fed after discharge, supporting the Baby Friendly Hospital Initiative recommendations for using cup feeding and avoiding bottle feeding when providing supplementation for preterm infants. The current study provides initial evidence for the implementation of cup feeding as a method of supplementation during hospitalization. If cup feeding is implemented in Egypt it will be necessary to first educate the medical and nursing staff. All healthcare personnel need to promote breastfeeding as the best and natural feeding method for all infants. Additionally, healthcare providers need to accept cup feeding as a safe, efficient feeding method and to learn safe cup feeding techniques. The finding that most mothers in both groups were able to initiate breastfeeding after discharge is interesting. Mothers did not have access to an electric breast pumps and did not provide breast milk to infants during hospitalization. Therefore, it is not known how mothers established and maintained their milk supply. These findings draw attention to the need for further exploration of the methods used by mothers of preterm infants to maintain their milk supply during infant hospitalization.

## Competing interests

The authors of this manuscript declare that they have no competing interests.

## Authors' contributions

AMA, the first author, carried out the study and wrote the manuscript. DAD supervised AMA in here pre-doctoral work and in her post-doctoral fellowship and worked closely with her preparing the manuscript. SAD, SRE, and IAS worked with the first author in the main study (preparing the proposal, data collection and data analysis).
